# Impact of Glomerular Filtration Rate on the Incidence and Prognosis of New-Onset Atrial Fibrillation in Acute Myocardial Infarction

**DOI:** 10.3390/jcm9051396

**Published:** 2020-05-09

**Authors:** Nicola Cosentino, Marco Ballarotto, Jeness Campodonico, Valentina Milazzo, Alice Bonomi, Simonetta Genovesi, Marco Moltrasio, Monica De Metrio, Mara Rubino, Fabrizio Veglia, Emilio Assanelli, Ivana Marana, Marco Grazi, Gianfranco Lauri, Antonio L. Bartorelli, Giancarlo Marenzi

**Affiliations:** 1 Centro Cardiologico Monzino, I.R.C.C.S., 20138 Milan, Italy; nicola.cosentino@ccfm.it (N.C.); m.ballarotto@gmail.com (M.B.); jeness.campodonico@ccfm.it (J.C.); valentina.milazzo@ccfm.it (V.M.); alice.bonomi@ccfm.it (A.B.); marco.moltrasio@ccfm.it (M.M.); monica.demetrio@ccfm.it (M.D.M.); mara.rubino@ccfm.it (M.R.); fabrizio.veglia@ccfm.it (F.V.); emilio.assanelli@ccfm.it (E.A.); ivana.marana@ccfm.it (I.M.); marco.grazi@ccfm.it (M.G.); gianfranco.lauri@ccfm.it (G.L.); antonio.bartorelli@ccfm.it (A.L.B.); 2 Nephrology Unit, San Gerardo Hospital, University of Milan-Bicocca, 20100 Milan, Italy; simonetta.genovesi@unimib.it; 3 Department of Biomedical and Clinical Sciences “Luigi Sacco”, University of Milan, 20157 Milan, Italy

**Keywords:** atrial fibrillation, acute myocardial infarction, renal function, mortality

## Abstract

Background: Atrial fibrillation (AF) is a frequent complication of acute myocardial infarction (AMI) and is associated with a worse prognosis. Patients with chronic kidney disease are more likely to develop AF. Whether the association between AF and glomerular filtration rate (GFR) is also true in AMI has never been investigated. Methods: We prospectively enrolled 2445 AMI patients. New-onset AF was recorded during hospitalization. Estimated GFR was estimated at admission, and patients were grouped according to their GFR (group 1 (*n* = 1887): GFR >60; group 2 (*n* = 492): GFR 60–30; group 3 (*n* = 66): GFR <30 mL/min/1.73 m^2^). The primary endpoint was AF incidence. In-hospital and long-term (median 5 years) mortality were secondary endpoints. Results: The AF incidence in the population was 10%, and it was 8%, 16%, 24% in groups 1, 2, 3, respectively (*p* < 0.0001). In the overall population, AF was associated with a higher in-hospital (5% vs. 1%; *p* < 0.0001) and long-term (34% vs. 13%; *p* < 0.0001) mortality. In each study group, in-hospital mortality was higher in AF patients (3.5% vs. 0.5%, 6.5% vs. 3.0%, 19% vs. 8%, respectively; *p* < 0.0001). A similar trend was observed for long-term mortality in three groups (20% vs. 9%, 51% vs. 24%, 81% vs. 50%; *p* < 0.0001). The higher risk of in-hospital and long-term mortality associated with AF in each group was confirmed after adjustment for major confounders. Conclusions: This study demonstrates that new-onset AF incidence during AMI, as well as the associated in-hospital and long-term mortality, increases in parallel with GFR reduction assessed at admission.

## 1. Introduction

Atrial fibrillation (AF) is the most common cardiac arrhythmia, and it often complicates acute myocardial infarction (AMI) [1,2,3,4,5,6,7,8]. Although current pharmacologic and mechanical therapeutic strategies have reduced the incidence of AF in this clinical setting, new-onset AF, defined as the arrhythmia occurring during the index event, still ranges between 6% and 12% in AMI patients [1,2,3,4,5,6,7,8]. A systematic review of the published literature demonstrated that new-onset AF in AMI has serious adverse implications regarding both in-hospital and long-term mortality [8]. This seems to prove correct for all AMI populations studied without significant differences regarding treatment and even for transient AF reversed to sinus rhythm before hospital discharge [8].

Patients with chronic kidney disease (CKD) are more likely to develop AF than the general population: The risk of AF progressively increases as renal function worsens [9,10]. Not only is the incidence of AF higher in CKD patients, but it also confers an increased risk of both stroke and overall mortality [9,10,11,12]. Potential explanations for the higher burden of AF in CKD include but are not limited to augmented sympathetic tone, activation of the renin-angiotensin-aldosterone system, and myocardial remodeling, mechanisms that are also enhanced during AMI. Nevertheless, very little is known about the incidence of new-onset AF, its correlates, and its association with short-term and long-term prognosis in AMI patients across glomerular filtration rate (GFR) values. Notably, high serum creatinine (sCr) concentration at hospital admission does not necessarily reflect the presence of CKD in AMI patients, but it may represent a variable combination of CKD and acute kidney injury due to the ongoing hemodynamic impairment [13].

In this study, we aimed to assess the incidence of new-onset AF according to GFR, estimated at hospital admission, and its relationship with short-term outcome and long-term all-cause mortality in a real-world cohort of AMI patients.

## 2. Methods

This was a prospective observational study. We enrolled all consecutive patients with AMI, both ST-elevation myocardial infarction (STEMI) and non-ST-elevation myocardial infarction (NSTEMI), admitted to the Intensive Cardiac Care Unit (ICCU) of Centro Cardiologico Monzino in Milan, Italy, between June 1, 2010, and May 31, 2018. Patients with permanent AF (*n* = 86) were excluded from the study. The study complied with the Declaration of Helsinki, and the Ethics Committee of our center approved the study (R519/CCM548). Written informed consent was obtained from all participants.

### 2.1. Study Protocol

Serum creatinine was measured at hospital admission in all patients, and GFR was estimated using the Modification of Diet in Renal Disease equation [14]. Patients were grouped according to their GFR value: GFR >60 mL/min/1.73 m^2^ (group 1), GFR 60–30 mL/min/1.73 m^2^ (group 2), and GFR <30 mL/min/1.73 m^2^ (group 3). All patients remained under continuous electrocardiographic monitoring during ICCU stay and at least for 72 h for those discharged from ICCU earlier. We considered any documented episode of AF lasting at least 30 min or requiring intervention because of symptoms or hemodynamic compromise. The treatment of AF was left to the discretion of the referring cardiologist. In particular, according to the internal clinical protocol, when AF episodes lasted more than 24 h despite prompt pharmacological therapy, electrical cardioversion was advised.

Study patients received medical treatment and coronary revascularization based on the current standards of care recommended by published guidelines on AMI [15]. Demographical, clinical and biochemical data, and echocardiographic left ventricular ejection fraction (LVEF) were collected at hospital admission. After hospital discharge, all patients were followed up for at least 1 year. 

The primary endpoint of the study was the incidence of new-onset AF during AMI hospitalization in the three study groups. In-hospital mortality and long-term (median 5 (1.2–8.5) years) all-cause mortality were the secondary endpoints of the study.

### 2.2. Statistical Analysis

A sample size of 2250 patients was calculated under the following assumptions: 10% overall incidence of AF (10), 70% of patients with GFR >60 mL/min/1.73 m^2^ [16], and an expected risk of AF (odds ratio (OR)) increasing by a factor of 2 from group 1 to group 2 and from group 2 to group 3. This sample size allowed a 90% statistical power in assessing a significant difference (α error of 0.05) of the primary endpoint between the three groups. Moreover, this sample size (*n* = 2250) allowed a 90% statistical power when an overall incidence of 20% of long-term all-cause mortality was considered [17], with an expected long-term mortality risk (hazard ratio (HR)) increasing by a factor of 1.3 from group 1 to group 2 and from group 2 to group 3. A total of 2400 patients represented the final sample size of our study, also considering a potential 7% of patients lacking data on the main variables of interest.

Continuous variables are presented as mean ± standard deviation. Variables with a skewed distribution are presented as median and interquartile ranges. Categorical data are presented as *n* (%). Trends across GFR groups were assessed by ANCOVA and by Mantel–Haenszel chi-square, as appropriate. The association between GFR groups, AF incidence, and study endpoints was assessed by logistic regression analysis. Results are presented as OR with 95% confidence intervals (CI). Cox proportional hazard model was also used to assess HR and 95% CI for long-term mortality associated with GFR groups.

Kaplan–Meier analysis was used to generate time-to-event curves for long-term mortality in patients with and without AF and stratified according to GFR groups. Log rank test was used to compare strata.

Analyses were adjusted for a model including independent predictors of each endpoint, identified by performing stepwise selection of variables.

All tests were 2-tailed, and a *p* < 0.05 was required for statistical significance. All analyses were performed using SAS version 9.4 (SAS Institute, Cary, NC, USA).

## 3. Results

In total, 2445 AMI patients (mean age 68 ± 12 years, 1806 men, 1148 STEMI) were enrolled in the study. Of them, 241 (10%) had new-onset AF (11% of STEMI and 8% of NSTEMI patients; *p* = 0.01). In 13 (5%) cases, electrical cardioversion was required, and 10 patients were discharged with persistent AF due to contraindication to cardioversion, treatment failure, or early AF recurrence. The baseline clinical characteristics of patients with and without AF are shown in Table 1.

Patients with AF were older and more likely to have comorbidities, prior cardiovascular events, and lower GFR and LVEF. Patients with AF experienced a higher rate of in-hospital complications, including mortality (Table 1).

One thousand eight hundred eighty-seven (77%) patients had an admission GFR >60 mL/min/1.73 m^2^ (group 1), 492 (20%) had GFR 60–30 mL/min/1.73 m^2^ (group 2), and 66 (3%) had GFR <30 mL/min/1.73 m^2^ (group 3). The baseline clinical characteristics and in-hospital complications of the three study groups are reported in Table 2.

**Figure 1:**
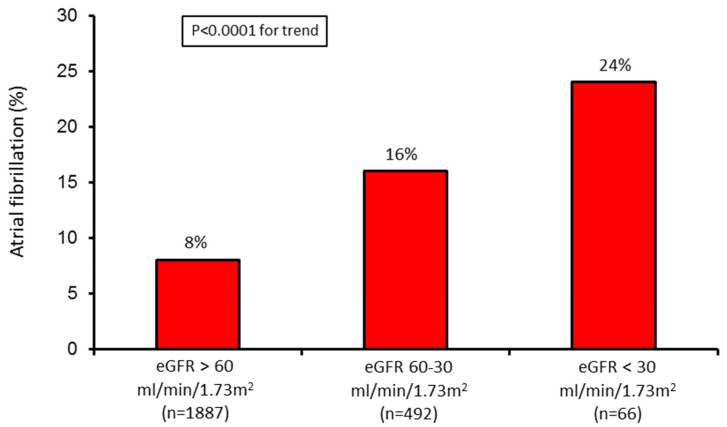
Incidence of atrial fibrillation (AF) in the three study groups. eGFR = estimated glomerular filtration rate.

The AF incidence increased in parallel with GFR reduction (Figure 1).

Table 3 shows the independent predictors of AF in the entire study population.

Atrial fibrillation was associated with an increased in-hospital mortality risk, even after adjustment for variables (age, AMI type, LVEF, GFR, Killip class, and admission glycemia) found to predict in-hospital mortality at stepwise analysis (adjusted OR 2.49; 95% CI 1.64–3.77; *p* < 0.0001).

**Figure 2:**
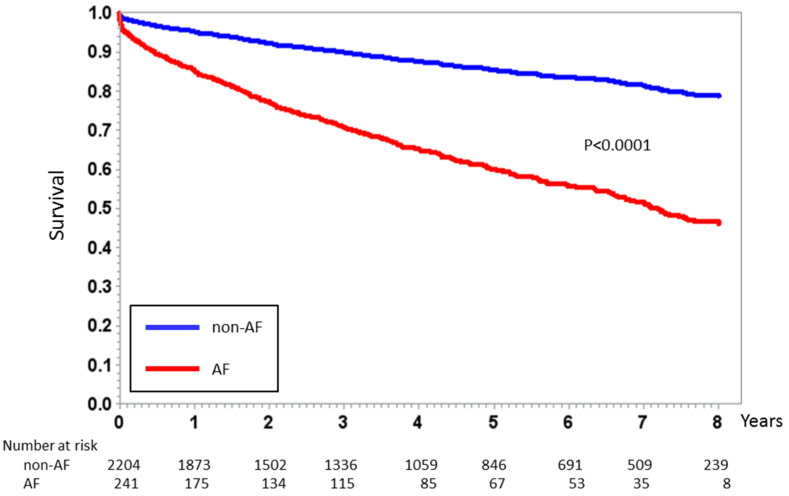
Kaplan–Meier curve analysis of all-cause mortality stratified according to new-onset AF occurrence during the index event in the entire study population. *p* value by Log rank test.

Similarly, long-term mortality was higher in patients experiencing AF during the index event (34% (*n* = 82) vs. 13% (*n* = 288); *p* < 0.0001) with an HR of 1.86 (95% CI 1.52–3.12; *p* < 0.0001), adjusted for independent predictors (age, diabetes mellitus, AMI type, prior AMI, LVEF, and GFR) of long-term mortality. Figure 2 shows the Kaplan–Meier curves for long-term mortality in patients with and without AF.

**Figure 3:**
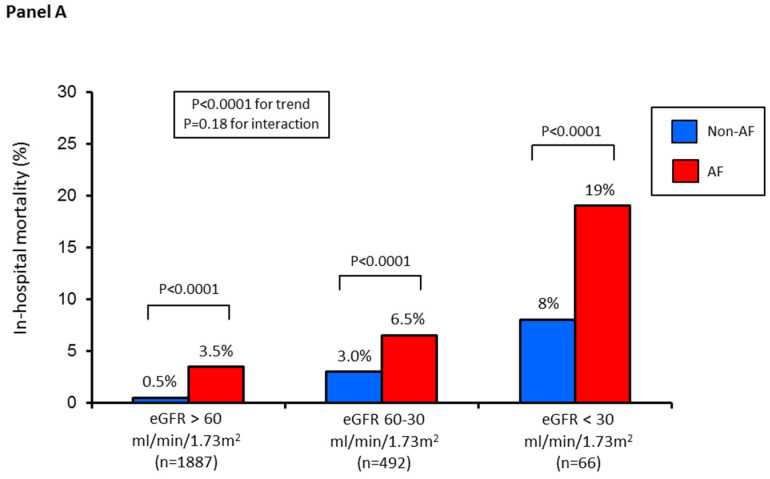

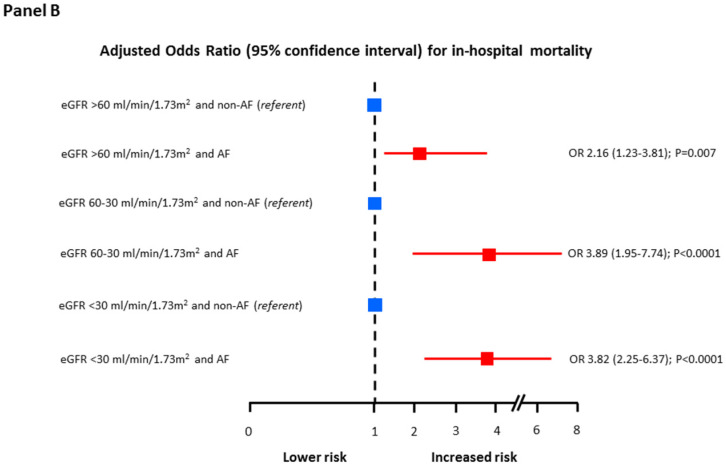
(**Panel A**) In-hospital mortality rate in the three study groups according to AF (yes vs. no). (**Panel B**) Adjusted odds ratio (OR) and 95% confidence intervals for in-hospital mortality according to AF in each study group. Odds ratios were adjusted for independent predictors of in-hospital mortality, identified by logistic regression analysis with stepwise selection of variables (age, acute myocardial infarction type, left ventricular ejection fraction, Killip class, and admission glycemia) in the entire study population.

In-hospital mortality increased progressively from group 1 to group 2 and to group 3 (1%, 4%, 11%, respectively; *p* < 0.0001 for trend). In the three groups, in-hospital mortality rate was significantly higher in patients with AF, and its onset was associated with an increased adjusted risk (Figure 3).

**Figure 4:**
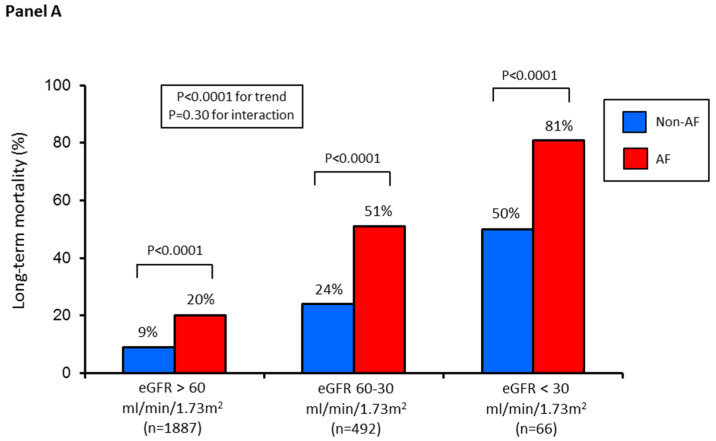

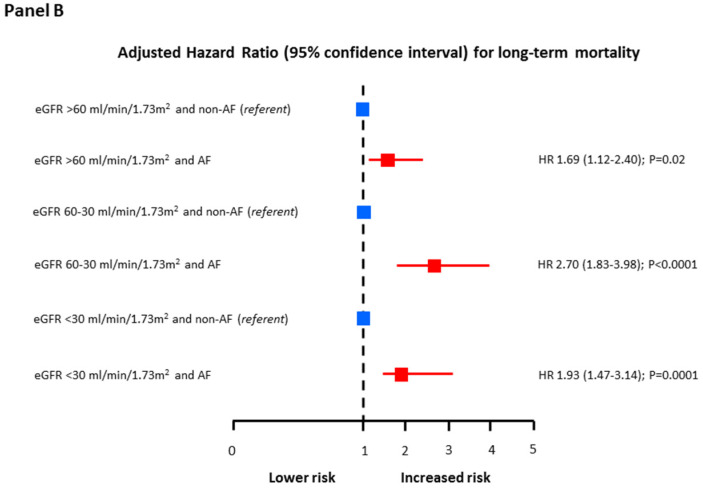
(**Panel A**) Long-term all-cause mortality rate in the three study groups according to AF (yes vs. no). In particular, the reported mortality rate refers to the events observed during the entire follow-up period (median 5 (1.2–8.5) years). (**Panel B**) Adjusted hazard ratios (HR) and 95% confidence intervals for long-term mortality according to AF in each study group. Hazard ratios were adjusted for independent predictors of long-term mortality, identified by logistic regression analysis with stepwise selection of variables (age, diabetes mellitus, acute myocardial infarction type, prior myocardial infarction, and left ventricular ejection fraction) in the entire study population.

Similarly, when considering the overall follow-up period (median 5 (1.2–8.5) years), long-term mortality increased progressively in the three groups (10%, 28%, 58%, respectively; *p* < 0.0001 for trend). Likewise, the incidence and adjusted risk of long-term mortality were higher in the three groups of patients with AF (Figure 4).

Table 4 shows the adjusted OR for in-hospital mortality and the adjusted HR for long-term mortality in the study population, according to GFR.

**Figure 5:**
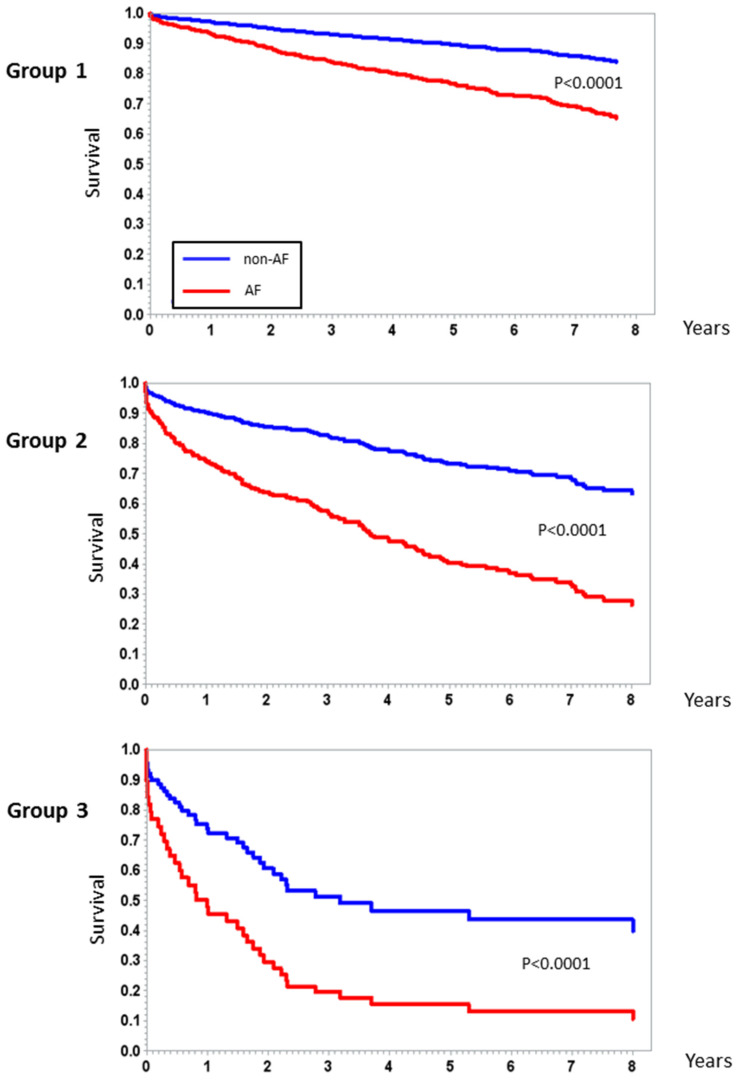
Kaplan–Meier curve analysis for long-term all-cause mortality stratified according to AF in the three study groups. *p* value by Log rank test.

The Kaplan–Meier curves for long-term mortality in patients with and without AF in the three study groups analyzed separately are shown in Figure 5.

## 4. Discussion

The major finding of the present study is that the incidence of new-onset AF during AMI, as well as its associated in-hospital and long-term mortality, increases in parallel with the severity of GFR reduction estimated at hospital admission.

Although current therapeutic strategies have reduced the incidence of AF in AMI patients, new-onset AF still occurs in a sizable number of patients with an incidence reported to vary between 6% and 12% [1,2,3,4,5,6,7,8,18,19,20]. Moreover, the bulk of the evidence demonstrates that AF onset in AMI patients has serious adverse prognostic implications regarding both in-hospital and long-term mortality [8,19]. This seems to apply to all AMI populations without significant differences according to their treatment [8]. On the other hand, several population-based studies evaluating the association between AF and CKD have consistently shown a higher prevalence of AF in CKD patients. Indeed, contemporary data show that about 20% of CKD patients have AF [9,10] and that the risk of AF increases as GFR declines [11,12]. Despite the evidence accumulated in the general population, no specific data are available regarding the relationship between AF and GFR in AMI. Notably, in this clinical setting, sCr value measured at hospital admission, from which GFR is usually estimated, may reflect a variable combination of pre-existing CKD and ongoing acute kidney injury due to hemodynamic instability associated with AMI. Moreover, when renal function acutely decreases, sCr increases slowly, usually within days, or may not even change until kidney function has decreased by about 50%. Thus, the well-known association between GFR and AF observed in the general population may not be directly transferred to AMI patients. Based on these premises, we performed a prospective observational study investigating the incidence of new-onset AF and its relationship with short-term and long-term mortality in a cohort of AMI patients across GFR values.

In our study, the incidence of AF was 10%, a value comparable to that reported in previous series of AMI patients [2,3,10,19]. Moreover, and similarly to previous studies, we found that patients experiencing AF during AMI were older, had more cardiovascular risk factors, and were more likely to have cardiac dysfunction [8,20,21]. As a result, the rate of in-hospital complications, including mortality, was higher than that of patients without AF. Moreover, the in-hospital prognostic power of AF was confirmed in our study also after adjustment for major predictors of in-hospital mortality in AMI, including Killip class, reinforcing the emerging concept that AF in itself can affect early prognosis [8,19,20,21,22]. Indeed, in a large database of 106,708 AMI patients [22], new-onset AF was associated with a 20% higher adjusted in-hospital mortality risk. In contrast, patients with AF at the time of hospital admission had a mortality rate that was not different from that of patients in sinus rhythm, presumably a reflection of persistent or chronic AF as opposed to de novo acute AF. Similarly, in The Optimal Trial in Myocardial Infarction with the Angiotensin II Antagonist Losartan (OPTIMAAL) trial [19], 30-day adjusted mortality risk was significantly higher (OR 3.83) in AMI patients who were admitted in sinus rhythm and developed AF during hospitalization than in those without this arrhythmia. Thus, the identification of other factors that are unique to patients with AF would be of interest.

Although a number of studies evaluated the clinical relevance of AF in AMI patients, to the best of our knowledge this is the first study that specifically investigated the impact of admission GFR on new-onset AF and their relationship in terms of short-term and long-term mortality. We found that the incidence and the risk of AF increase as GFR declines, even after adjustment for major clinical predictors. Moreover, AF patients showed a significant increase in hospital mortality, as compared to their non-AF counterparts, at every GFR level. Again, this was true also when major cofounders were considered, including Killip class. The mechanisms underlying the close association between GFR, AF, and in-hospital clinical outcome during AMI are unclear. However, some speculations can be suggested. Patients with AMI and low GFR are more vulnerable to the acute hemodynamic impairment that has been linked to the development of AF, such as increase in left atrial pressure and volume and left ventricular diastolic dysfunction [23]. Furthermore, disturbances of the reflex autonomic control of heart rate have been associated with a doubling of the likelihood of new AF occurrence in patients with AMI [24]. In this regard, sympathetic over-activity has been demonstrated to occur in both CKD and acute kidney injury, and to be directly associated with the severity of renal failure [25,26]. Recently, inflammation, which is a typical feature of renal insufficiency, has also been shown to be independently associated with AF onset in AMI patients [27]. In agreement with these observations, our study showed that the levels of high-sensitivity C-reactive protein at hospital admission were significantly higher in patients with AF. Taken together, these findings suggest a greater electrical vulnerability, in terms of AF risk, in patients with low GFR also during AMI, which may possibly contribute to worsen clinical prognosis.

We also confirmed the adverse impact of AF during AMI on long-term mortality, even after adjustment for the most relevant prognostic variables [2,3,18,19]. To date, the longest follow-up has been performed in the OPTIMAAL trial, in which the hazard linked to AF in the setting of AMI was assessed over a 3-year period [18]. The authors found that new-onset AF is associated with an almost double mortality risk. Our data further extend these findings, by showing a persistent greater all-cause mortality risk associated with AF in all study groups up to a median follow-up of 5 years. In addition to the well-known negative impact of low GFR on long-term mortality in AMI, even a single episode of AF seems to worsen the prognosis associated with each GFR level. Therefore, the detection of AF episodes during hospitalization, in addition to an early assessment of GFR, allows a more accurate risk stratification of AMI patients. The mechanisms underlying this association cannot be inferred from our data. However, there is recent evidence that AF and CKD may contribute to increased long-term mortality via pro-coagulant and inflammatory pathways [28,29]. Notably, these two conditions are associated with increased levels of C-reactive protein, interleukin-6, plasmin-antiplasmin complex, factor VII, and factor VIII, which by inducing inflammation and hypercoagulability may further promote atherosclerosis progression and thrombosis [28,29].

Our study may have some potential clinical implications. Prolonged continuous electrocardiographic monitoring may be useful in AMI patients, particularly in those with reduced GFR, to detect transient and asymptomatic AF episodes. Indeed, most AF events in AMI patients are asymptomatic, probably because of the high frequency of beta-blocker treatment [20]. In high-risk patients, the potential benefit associated with therapeutic strategies directed towards a reduction of in-hospital AF burden should be investigated, with the ultimate goal of improving prognosis. Moreover, a closer clinical follow-up of these patients should be considered.

The strengths of our study include a large and well-characterized AMI population, adjustment for major risk factors, a special focus on the association between new-onset AF and GFR, and a very long-term follow-up. However, some limitations should be acknowledged. First, unmeasured confounding factors cannot be excluded, and we cannot provide concrete proof of a cause-effect relationship between low GFR and new-onset AF. Second, most AMI patients underwent emergent or urgent percutaneous coronary intervention. In our study, myocardial revascularization was associated with a lower AF risk; this may have influenced our results, and their overall validity in all AMI patients needs to be evaluated. Third, the impact on outcomes of AF timing of onset, its duration, and its recurrence after hospital discharge was not investigated. Finally, we considered only all-cause mortality at long-term follow-up. Therefore, we were unable to evaluate cause-specific mortality, particularly in patients with AF and in those with low GFR.

## 5. Conclusions

In conclusion, our study demonstrates that the incidence of new-onset AF during the acute phase of AMI increases in parallel with the severity of GFR reduction at hospital admission. New-onset AF is adversely associated with short-term and long-term prognosis, with a negative effect that is additional to that of GFR across all stages of its reduction.

## Figures and Tables

**Table 1:** Baseline characteristics and in-hospital complications of the study patients according to the occurrence of atrial fibrillation.

Variable	Atrial Fibrillation		*p* Value
	No (*n* = 2204)	Yes (*n* = 241)	
Age (years)	66 ± 12	75 ± 10	<0.0001
Male sex, *n* (%)	1647 (75%)	159 (66%)	0.003
Body weight (kg)	76 ± 14	74 ± 16	0.01
Diabetes mellitus, *n* (%)	467 (21%)	81 (34%)	<0.0001
Hypertension, *n* (%)	1398 (63%)	194 (80%)	<0.0001
Smokers, *n* (%)	1191 (54%)	98 (31%)	<0.0001
Hyperlipidemia, *n* (%)	1105 (50%)	123 (51%)	0.80
Prior myocardial infarction, *n* (%)	571 (26%)	77 (32%)	0.004
Prior CABG, *n* (%)	255 (12%)	39 (16%)	0.03
Prior PCI, *n* (%)	578 (26%)	68 (28%)	0.48
Left ventricular ejection fraction (%)	51 ± 11	43 ± 13	<0.0001
STEMI, *n* (%)	1017 (46%)	131 (54%)	0.01
CA/PCI during hospitalization, *n* (%)	2080 (95%)	210 (87%)	<0.0001
**Laboratory values at hospital admission**			
Serum creatinine (mg/dL)	1.02 ± 0.44	1.23 ± 0.82	<0.0001
eGFR (mL/min/1.73 m^2^)	79 ± 26	68 ± 26	<0.0001
Hemoglobin (g/dL)	13.8 ± 1.8	13.2 ± 1.9	<0.0001
Blood glucose (mg/dL)	147 ± 60	166 ± 72	<0.0001
hs-TnI (ng/L)	400 (70–2340)	1064 (130–6690)	0.0001 *
hs-CRP (mg/L)	3.39 (1.37–10.71)	7.06 (2.12–36.41)	<0.0001 *
**Medication before hospital admission**			
Aspirin, *n* (%)	822 (37%)	100 (41%)	0.2
Statins, *n* (%)	747 (34%)	99 (42%)	0.02
Beta-blockers, *n* (%)	772 (35%)	104 (43%)	0.001
ACE/AR blockers, *n* (%)	859 (39%)	121 (50%)	0.001
**In-hospital complications**			
In-hospital death, *n* (%)	26 (1.2%)	13 (5.4%)	<0.0001
Cardiogenic shock, *n* (%)	86 (4%)	35 (15%)	<0.0001
Acute pulmonary edema, *n* (%)	150 (7%)	76 (32%)	<0.0001
Mechanical ventilation, *n* (%)	55 (3%)	28 (12%)	<0.0001
VT/VF, *n* (%)	147 (7%)	32 (13%)	0.0001
High-degree AV block, *n* (%)	69 (3%)	18 (7%)	0.0001
Major bleeding, *n* (%)	50 (2%)	26 (11%)	<0.0001
hs-TnI peak value (ng/L)	37,442 ± 77,002	74,162 ± 168,968	<0.0001
**Medication at hospital discharge** #			
Dual antiplatelet therapy, *n* (%)	2014 (92%)	209 (92%)	0.66
Statins, *n* (%)	2005 (92%)	207 (91%)	0.50
Beta-blockers, *n* (%)	1702 (78%)	175 (77%)	0.63
ACE/AR blockers, *n* (%)	1401 (64%)	133 (58%)	0.07
Oral anticoagulant			
therapy, *n* (%)	48 (2%)	30 (13%)	<0.0001

ACE = angiotensin-converting enzyme; AR = angiotensin II receptor; AV = atrio-ventricular; CA = coronary angiography; CABG = coronary artery bypass graft; eGFR = estimated glomerular filtration rate; hs-CRP = high-sensitivity C-reactive protein; hs-TnI = high-sensitivity troponin I; PCI = percutaneous coronary intervention; STEMI = ST-segment elevation myocardial infarction; VF = ventricular fibrillation; VT = ventricular tachycardia. * by Wilcoxon Rank-Sum test. # Percentages are calculated on the number of discharged patients.

**Table 2:** Baseline characteristics and in-hospital complications in the three study groups.

Variable	Group 1 (*n* = 1887)	Group 2 (*n* = 492)	Group 3 (*n* = 66)	*p* Value (for Trend)
Age (years)	65 ± 12	73 ± 11	75 ± 9	<0.0001
Male sex, *n* (%)	1450 (77%)	319 (65%)	37 (56%)	<0.0001
Body weight (kg)	76 ± 14	76 ± 16	73 ± 12	0.69
Diabetes mellitus, *n* (%)	354 (19%)	162 (33%)	32 (48%)	<0.0001
Hypertension, n (%)	1148 (61%)	387 (79%)	57 (87%)	<0.0001
Smokers, *n* (%)	1068 (57%)	198 (40%)	23 (36%)	<0.0001
Hyperlipidemia, *n* (%)	917 (49%)	268 (54%)	43 (65%)	0.0009
Prior myocardial infarction, *n* (%)	431 (23%)	185 (38%)	32 (48%)	<0.0001
Prior CABG, *n* (%)	188 (10%)	90 (18%)	16 (24%)	<0.0001
Prior PCI, *n* (%)	433 (23%)	188 (38%)	25 (38%)	<0.0001
Left ventricular ejection fraction (%)	51 ± 11	48 ± 13	43 ± 14	<0.0001
STEMI, *n* (%)	915 (48%)	211 (43%)	22 (33%)	0.002
CA/PCI during hospitalization, *n* (%)	1796 (95%)	439 (89%)	55 (83%)	<0.0001
**Laboratory values at hospital admission**				
Serum creatinine (mg/dL)	0.88 ± 0.18	1.39 ± 0.31	3.01 ± 1.370	<0.0001
eGFR (mL/min/1.73 m^2^)	88 ± 21	48 ± 8	21 ± 5	<0.0001
Hemoglobin (g/dL)	14.0 ± 1.7	13.1 ± 1.9	11.3 ± 1.9	<0.0001
Blood glucose (mg/dL)	143 ± 56	167 ± 70	193 ± 92	<0.0001
hs-TnI (ng/L)	430 (76–2460)	440 (80–2700)	1087 (80–5024)	0.12
**Medication before hospital admission**				
Aspirin, *n* (%)	638 (34%)	242 (49%)	42 (64%)	<0.0001
Statins, *n* (%)	587 (31%)	223 (46%)	36 (55%)	<0.0001
Beta-blockers, *n* (%)	604 (32%)	229 (47%)	43 (65%)	<0.0001
ACE/AR blockers, *n* (%)	683 (36%)	271 (55%)	26 (39%)	<0.0001
**In-hospital complications**				
In-hospital death, *n* (%)	14 (0.7%)	18 (4%)	7 (11%)	<0.0001
Cardiogenic shock, *n* (%)	66 (3%)	44 (9%)	11 (17)	<0.0001
Acute pulmonary edema, *n* (%)	118 (6%)	84 (17%)	24 (36%)	<0.0001
Mechanical ventilation, *n* (%)	37 (2%)	38 (8%)	8 (12%)	<0.0001
VT/VF, *n* (%)	128 (7%)	46 (9%)	5 (8%)	0.11
High-degree AV block, *n* (%)	50 (3%)	34 (7%)	3 (5%)	0.001
Major bleeding, *n* (%)	41 (2%)	26 (5%)	9 (14%)	<0.0001
hs-TnI peak value (ng/L)	37,001 ± 749,442	49,503 ± 108,275	71,774 ± 240,209	0.65

**Table 3:** Adjusted odds ratio (OR) and 95% confidence intervals (CI) for independent predictors of atrial fibrillation found at stepwise analysis in the overall study population.

Variable	OR * (95% CI)	*p* Value
eGFR 60–30 vs. >60 mL/min/1.73 m^2^	2.28 (1.70–3.06)	<0.0001
eGFR <30 vs. >60 mL/min/1.73 m^2^	3.81 (2.12–6.87)	<0.0001
STEMI vs. NSTEMI	1.48 (1.23–3.93)	0.0001
Age (every 10-year increase)	2.12 (1.38–3.66)	<0.0001
LVEF (every 10% decrease)	1.64 (1.37–2.97)	<0.0001
Killip class III-IV vs. I-II	1.98 (1.65–3.13)	<0.0001
PCI (no vs. yes)	1.73 (1.31–3.01)	<0.0001

eGFR = estimated glomerular filtration rate; LVEF = left ventricular; NSTEMI = non-ST-elevation myocardial infarction; * OR were adjusted for each other.

**Table 4:** Adjusted OR for in-hospital mortality and hazard ratio (HR) for long-term mortality between study groups.

**Variable**	**Adjusted OR (95% CI)**	***p* Value**
eGFR 60–30 vs. >60 mL/min/1.73 m^2^	2.69 (1.13–6.37)	0.003
eGFR <30 vs. 60–30 mL/min/1.73 m^2^	2.53 (1.35–7.68)	0.0001
**Variable**	**Adjusted HR (95% CI)**	***p* Value**
eGFR 60–30 vs. >60 mL/min/1.73 m^2^	1.44 (1.14–1.81)	0.002
eGFR <30 vs. 60–30 mL/min/1.73 m^2^	2.24 (1.55–3.23)	<0.0001

CI = 95% confidence intervals; eGFR = estimated glomerular filtration rate.
